# Knowledge, Attitudes, and Practices of Iodized Salt Among Adults Aged 18 to 65 in the United Arab Emirates

**DOI:** 10.7759/cureus.55844

**Published:** 2024-03-09

**Authors:** Leen Salma, Tasnim Musameh, Adham Motawea, Ahmed Elshafiey, Khulood Al Ketbi, Amna Alkindi, Wafa Alnakhi

**Affiliations:** 1 College of Medicine, University of Sharjah, Sharjah, ARE; 2 Department of Family and Community Medicine and Behavioral Sciences, University of Sharjah, Sharjah, ARE

**Keywords:** practices, attitudes, knowledge, iodine deficiency, iodine, iodized salt

## Abstract

Background: Iodine plays a big role in growth and development and is used in the synthesis of thyroid hormones that regulate body organ functions. Its most important source is iodized salt. Iodine deficiency impairs cognitive development and precipitates many thyroid diseases.

Aim: The purpose of our study is to measure the knowledge, attitudes, and practices towards iodized salt in adults aged 18-65 years in the United Arab Emirates.

Methods: This is a cross-sectional study that included 406 participants. A self-administered questionnaire was distributed in public places across the Emirates. The survey included the socio-demographic characteristics of the participants with questions related to knowledge, attitudes, and practices towards iodized salt. A knowledge score out of 13 was calculated. Chi-square, Mann-Whitney U, and Independent T-tests were used for analysis on the Statistical Package for the Social Sciences (IBM SPSS Statistics for Windows, IBM Corp., Version 22.0, Armonk, NY), with a p-value less than 5% considered statistically significant.

Results: Out of the 406 respondents, 60% (n=246) were between the ages of 18-34 years and 71% (n=287) were at university level. The mean knowledge score was higher in participants with higher educational levels compared to participants with lower education levels (4.30 vs 2.63, respectively) and in older ages (35-65 years) compared to younger ages (18-34 years) (4.20 vs 3.59, respectively). Almost half of the participants buy iodized salt (48%, n=196), a minority buy non-iodized salt (14%, n=55), and the rest do not know what type of salt they buy. Most of the participants who use iodized salt store it in a closed container and do not expose it to sunlight.

Conclusion: Knowledge about iodized salt is relatively low. Less than half of the participants use it, while most of the participants who use iodized salt follow the right storage recommendations. Our study suggests the implementation of public campaigns to raise awareness about the importance of iodized salt and the benefits of using it.

## Introduction

Iodine, an essential micronutrient that is crucial for the synthesis of thyroid hormones and optimal functioning of the thyroid gland, also plays a pivotal role in maintaining optimal health throughout the lifespan. Its significance comes from its impact on thyroid function, cognitive development, and overall well-being [1]. The issue of iodine deficiency is a global concern, with approximately 2 billion people experiencing iodine deficiency, and around 50 million displaying clinical manifestations [1]. Women in their reproductive period are particularly vulnerable, as their children face a risk of developing complications related to iodine deficiency [2]. Notably, the global landscape is evolving with the advent of fortified foods, public health campaigns, increased awareness, and most importantly, the widespread use of iodized salt. By the year 2020, around 124 countries mandated salt iodization, and another 21 countries allowed voluntary iodization, resulting in 88% of the global population using iodized salt [3]. Particularly noteworthy is the effectiveness of this approach in reducing iodine deficiency disorders in the Eastern Mediterranean region, as indicated by the World Health Organization (WHO) [4].

In the United Arab Emirates (UAE), a nation marked by remarkable economic growth and cultural diversity, the intricate relationship between iodine intake and public health has become an area of increasing significance. A study conducted by the Ministry of Health in 2009 revealed that around 94.1% of families in the UAE use iodized salt. This marks a significant improvement compared to a previous study conducted in 1999, which found that only 6.5% of families used iodized salt and 67% of UAE children did not consume sufficient amounts of iodine-rich food [5]. Nonetheless, there is still a deficiency in recent, comprehensive research assessing the knowledge level regarding the utilization of iodized salt in the UAE.

Iodized salt defined as regular salt that has been fortified with either potassium iodate, potassium iodide, sodium iodate, or sodium iodide is a simple yet effective public health intervention; its adoption, however, hinges on multifaceted factors such as public awareness, cultural beliefs, and individual dietary habits. Understanding the current state of knowledge, attitudes, and practices related to iodized salt in the UAE is paramount for designing targeted interventions and ensuring the sustained well-being of the population.

This study aimed to measure the knowledge, attitudes, and practices towards iodized salt in adults aged 18-65 years in the UAE. We hypothesize that older individuals, having had more exposure to health-related information over time and being the main providers in the households, may possess a higher degree of knowledge about the benefits and usage of iodized salt. Additionally, we anticipate that individuals with higher academic levels may exhibit greater awareness due to their access to formal education and information resources. In this paper, we present the objectives, methods, and findings of our investigation, aiming to contribute valuable insights that can inform public health initiatives and educational strategies in the UAE.

We participated in the 10th International Family Medicine Conference held in Dubai in November 2023, where our research abstract was accepted for presentation, allowing us to deliver an oral presentation and showcase our poster.

## Materials and methods

Study design, setting, and participants

The study used a cross-sectional research design with convenience sampling and a non-probability approach. The questionnaire was self-administered and distributed to adults from ages 18 to 65 years in public venues throughout the UAE in February 2020. The public places that were targeted included universities, parks, malls, and workplaces. The questionnaire had versions in Arabic and English and respondents were given 7 to 10 minutes which is the time allocated for survey completion.

The sample size was determined using the formula: n = 4p(1-p)/SE^2^ assuming a 5% margin of error (ME) and a 50% prevalence (P). The resultant sample size was 385, rounded to the nearest hundred, yielding a final sample size of 400. Ultimately, we had 406 respondents participate in the study.

Inclusion criteria encompassed adults aged 18 to 65 who understand either Arabic or English. Meanwhile, participants with specific demographic subsets, including individuals involved in medical-related occupations or educational pursuits, as well as those experiencing mental disabilities were excluded from the study. These criteria were established to ensure the selected sample population aligned precisely with the research's intended focus.

Ethics approval

Ethical clearance was obtained from the Research Ethics Committee at the University of Sharjah (Reference number: REC-20-01-30-04-S).

Measures

Data collection employed a structured questionnaire, designed based on a thorough literature review and adapted from comparable studies. The research incorporated four sections, comprising a total of 30 questions. Out of the 30 questions, eight questions pertained to demographics, 13 questions for knowledge, four questions for attitudes, and five questions for practices. All queries were closed-ended to streamline completion and minimize the required time. For questions related to knowledge, attitudes, and practices that can be quantified on a scale, Likert scales were employed. The knowledge of both iodine and iodized salt was measured using five questions from the questionnaire and calculated as a score from 0 to 13. Participants scoring 7 or higher were categorized as possessing good knowledge, while those scoring below 7 were classified as having poor knowledge. The mean knowledge score was then compared between two age groups (18-34 and 35-65) and two educational levels (below university level and at university level or higher).

Statistical analysis

Data was encoded, inputted, and analyzed using the Statistical Package for the Social Sciences (IBM SPSS Statistics for Windows, IBM Corp., Version 22.0, Armonk, NY). Descriptive statistics, including measures for data summarization such as frequency and relative frequency, central tendency (mean, median, mode), and variability (standard deviation), were employed as suitable for the data type. Bivariate analysis was conducted to examine variable relationships. Inferential statistical tests, encompassing the Chi-square test, Mann-Whitney U test, and Independent T-test were applied as appropriate. An established p-value <5% was considered as statistically significant.

## Results

Socio-demographics of study participants

The study involved 406 participants with almost equal proportions of males and females (females 51.7%, n=210; males 47.5%, n=193). Participants were mainly young adults with 82.3% (n=334) of participants being under the age of 45. The most prevalent age group in the study was the 18 to 24 age group (33.3%, n=135). As for the occupational status, half of the participants were full-time employees (52.7%, n=214). Most of the participants completed university-level education (70.7%, n=287). Most of the participants were residing in Sharjah (41.4%, n=168), followed by Dubai (21.9%, n=89). In terms of marital status, half of the participants reported being married (52.2%, n=212). In response to inquiries about cooking frequency, 21.4% (n=87) of participants indicated that they 'never' cook, while 20.7% (n=84) reported they 'always' cook. Additionally, 33.5% (n=136) of respondents reported that more than five people live in their household, as shown in Table 1.

**Table 1:** Socio-demographics of study participants (n=406)

Characteristics	(n)	(%)
Gender		
Male	193	47.5%
Female	210	51.7%
Age (Years)		
18-24	135	33.3%
25-34	111	27.3%
35-44	88	21.7%
45-54	46	11.3%
55-65	24	5.9%
Occupational status		
Unemployed	121	29.8%
Part-time employment	31	7.6%
Full-time employment	214	52.7%
Housewife	24	5.9%
Retired	14	3.4%
Education Level		
Didn’t complete elementary	4	1%
Elementary (Grades 1- 6)	8	2%
Intermediate (Grades 7- 9)	4	1%
Secondary (Grades 10-12)	102	25.1%
University	287	70.7%
Emirates of residency		
Abu Dhabi	46	11.3%
Dubai	89	21.9%
Sharjah	168	41.4%
Ajman	53	13.1%
Umm Al Quwain	6	1.5%
Ras Al Khaimah	7	1.7%
Fujairah	36	8.9%
Marital Status		
Married	212	52.2%
Single	191	47%
Cooking frequency		
Always	84	20.7%
Usually (5-6 days/week)	36	8.9%
Often (3-4 days/week)	61	15%
Sometimes (1-2 days/week)	63	15.5%
Rarely (1-2 days/month)	72	17.7%
Never	87	21.4%
No. of residents in the household		
Living Alone	24	5.9%
Two people	39	9.6%
Three people	51	12.6%
Four people	68	16.7%
Five people	86	21.2%
More than five people	136	33.5%

Knowledge of participants on iodine

The majority of the study participants have heard about iodine before. Among those who were aware of iodine, seafood was believed to be the main source by 54.2% (n=220) of participants. The thyroid gland was identified as the organ that requires iodine for functioning by 53% (n=153) of participants, followed by the brain by 14% (n=39). The consequences of iodine deficiency were perceived to be physical damage (27.8%, n=113) and slow growth (21.4%, n=87), while mental damage was the least chosen (16.3%, n=66). A considerable portion of participants (25.1%, n=102) did not know the consequences of iodine deficiency. These findings are summarized in Table 2.

**Table 2:** Knowledge about iodine

Questions	Frequency	(%)
Have you ever heard of iodine?		
Yes	308	75.9%
No	97	23.9%
Which foods do you think contain iodine?		
Fruits and vegetables	51	12.6%
Seafood	220	54.2%
Meat and poultry	39	9.6%
Dairy products	75	18.5%
I don’t know	38	9.4%
Do you know which part of the body needs iodine for proper function?		
Yes	206	50.7%
No	102	25.1%
If (Yes) which body part needs iodine to function?		
Brain	39	14%
Muscles	31	11%
Thyroid gland	153	53%
Digestive system	23	8%
Heart	21	7%
Lungs	13	5%
What do you think are the consequences of having low levels of iodine in your body?		
Slow growth	87	21.4%
Mental damage	66	16.3%
Physical damage	113	27.8%
I don’t know	102	25.1%

Knowledge of participants on iodized salt

A total of 50% (n=203) of the participants believed they knew what iodized salt was in comparison to 22.9% (n=93) who did not know what iodized salt was. About 65.6% (n=266) of the participants reported that iodized salt is better than regular salt (non-iodized) with 25.9% (n=105) stating that it is much better while 25.4% (n=103) stated that they're about the same benefit. A total of 58.6% (n=238) reported they think that iodized salt is beneficial, and those who reported iodized salt is beneficial believed it is beneficial in preventing thyroid problems (29.3%, n=159). About 40% (n=164) of the participants did not know how much iodized salt should be consumed in comparison to regular salt (non-iodized). When asked about the prevention of iodine deficiency, 35.6% (n=190) of participants stated that low levels of iodine can be prevented through consuming seafood. Around 41.6% (n=169) believed that iodized salt is worth its price. Regarding the importance of iodized salt in their diet, 20.4% (n=83) stated that it is very important (Table 3).

**Table 3:** Knowledge about iodized salt

Questions	Frequency	(%)
Do you know what iodized salt is?		
Yes	203	50%
No	93	22.9%
I am not sure	109	26.8%
Do you think iodized salt is better than regular salt?		
Much worse	8	2%
Somewhat worse	15	3.7%
About the same	103	25.4%
Somewhat better	161	39.7%
Much better	105	25.9%
Do you think iodized salt is beneficial?		
Yes	238	58.6%
No	40	9.9%
I don’t know	126	31%
If (yes) what are the benefits of iodized salt?		
Prevents thyroid problems	159	29.3%
Improves overall health	115	21.2%
Improves pregnancy outcomes	31	5.7%
Mental and physical growth	100	18.4%
I don’t know	125	23%
I don’t think it’s beneficial	11	2%
How much iodized salt do you think should be consumed in comparison to regular salt?		
Iodized salt should be consumed more	69	17%
Iodized salt should be consumed less	67	16.5%
Iodized salt should be consumed in similar amounts of regular salt	103	25.4%
I don’t know	164	40.4%
How do you think you can prevent low levels of iodine in your body?		
Iodized salt	143	26.8%
Iodine supplements	94	17.6%
Seafood	190	35.6%
I don’t know	105	20%
Do you think iodized salt is worth its price?		
Yes	169	41.6%
No	61	15%
I don’t know	174	42.9%
How important do you think iodized salt is in your diet?		
Not at all important	42	10.3%
Low importance	35	8.6%
Slightly important	77	19%
Neutral	70	17.2%
Moderately important	81	20%
Very important	83	20.4%
Extremely important	17	4.2%

Statistical analysis of knowledge levels regarding iodine and iodized salt across sociodemographic characteristics

**Figure 1:**
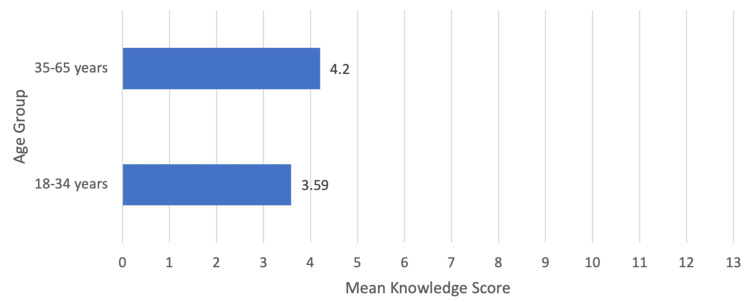
Relationship between mean knowledge score and age group There is a higher mean knowledge score (4.20 ± 3.040) in participants aged 35-65 (N=158, 39%) than in those aged 18-34 (N=246, 61%) with a mean knowledge score of (3.59 ± 2.918). Mann-Whitney U test revealed a p-value= 0.025*. *p-value <0.05 is considered statistically significant.

**Figure 2:**
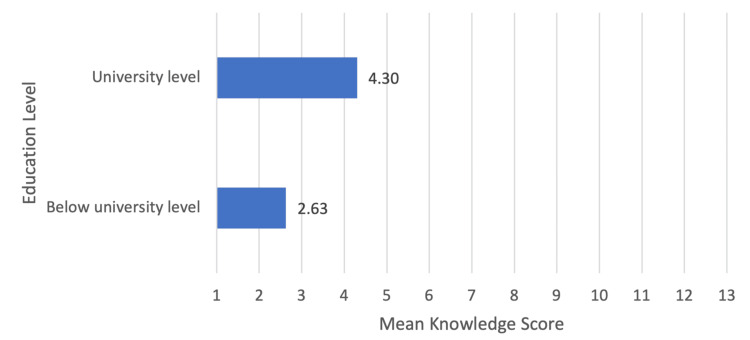
Relationship between mean knowledge score and educational level There is a higher knowledge score (4.30 ± 2.995) in participants who are at or completed university-level education (N=287, 71%) in comparison to those with a degree below university level (N=118, 29%) with a mean knowledge score of (2.63 ± 2.595). Independent T-test revealed a significant difference (p-value <0.001)*. *p-value <0.05 considered statistically significant.

Statistical analysis was performed to compare the knowledge of iodine and the knowledge of iodized salt with the sociodemographic characteristics. By looking at the relation between the knowledge about iodine and the level of education, it was found to be very significant (P-value < 0.0005), which was the same for the relationship between knowing iodized salt and buying it. Moreover, the relationship between level of education and knowledge about iodized salt was significant as well (P-value <0.001), and so was the relationship between age and knowledge about iodized salt (P-value = 0.025). However, there was no relationship between age and knowledge about iodine. The mean knowledge scores are shown in Figures 1-2.

The participants' knowledge scores were determined by their responses to a set of questions from the knowledge section. Each participant received a score out of 13. Correct answers included knowing that both seafood and dairy products are a major source of iodine, identifying that the thyroid gland is the body part requiring iodine to function, and understanding the consequences of low iodine levels including slow growth and mental/physical damage. Moreover, acknowledges the benefits of iodized salt in preventing thyroid problems, improving overall health, improving pregnancy outcomes, and also improving physical and mental growth. Finally, knowing how to prevent low iodine levels in the body, by using iodized salt, taking iodine supplements, and consuming seafood. Our findings revealed that 20.4% (n=83) exhibited good knowledge levels, while 79.5% (n=323) participants demonstrated poor knowledge levels.

Attitudes of participants on iodized salt

As shown in Table 4, about half of the participants (48.3%, n=196) buy iodized salt, and the majority believe that it does have health benefits (31.3%, n=127). Some people (10.3% n=42) buy iodized salt because they were advised to do so. A small portion of participants (13.5%, n=55) stated that they do not buy iodized salt. The majority of respondents that do not buy iodized salt stated that the reason behind that is that they did not know the importance of iodized salt (63%, n=35) and 56.4% (n=229) of the participants showed that they had no concerns about adding iodized salt to their diet.

**Table 4:** Attitudes about iodized salt

Questions	Frequency	(%)
What kind of salt do you buy?		
Iodized salt	196	48.3%
Non-iodized salt	55	13.5%
I don’t know	155	38.2%
If yes, why would you buy iodized salt?		
I know it’s beneficial	127	31.3%
Other kinds of salt aren’t available	12	3%
I was advised to buy it	42	10.3%
If not, why would you not buy iodized salt?		
I don’t know why it is important	35	63%
It is more expensive	8	14%
It is less salty	2	0.5%
It is harmful	3	0.7%
Do you have any concerns about adding iodized salt to your diet?		
Yes	75	18.5%
No	229	56.4%
I’m not sure	100	24.6%

Practices of participants towards iodized salt

When asked about iodized salt usage, a third of the participants (33%, n=134) denied using iodized salt or were not sure if they used iodized salt in the first place. Those who always use it were about 25.6% (n=104). When questioned about the practices regarding storage of iodized salt, about a third of the participants (31.3%, n=127) were not sure about the duration of storage and only 19% (n=77) of participants stored their salt for a period of less than two months. In addition, 37.9% (n=154) store iodized salt in cabinets, and about half of the participants (51.5%, n=209) store iodized salt away from sunlight. Similarly, about half of the participants (52%, n=211) store their iodized salt in a closed container. As reported in Table 5, participants who do not buy or use iodized salt were exempted from answering questions on storage practices of iodized salt.

**Table 5:** Practices of iodized salt

Questions	Frequency	(%)
How often do you use iodized salt?		
Always	104	25.6%
Usually (5-6 days/week)	31	7.6%
Often (3-4 days/week)	46	11.3%
Sometimes (1-2 days/week)	29	7.1%
Rarely (1-2 days/month)	60	14.8%
Never/I don’t know if I use iodized salt	134	33%
For how long do you store iodized salt?		
Less than two months	77	19%
More than two months	64	15.8%
I’m not sure	127	31.3%
Where do you store iodized salt?		
Cabinets	154	37.9%
Drawer	73	18%
Fridge	14	3.4%
Outside the kitchen	3	0.7%
I’m not sure	22	5.4%
Is the storage place exposed to sunlight?		
Yes	29	7.1%
No	209	51.5%
I’m not sure	30	7.4%
Do you store it in a closed container?		
Yes	211	52%
No	38	9.4%
I’m not sure	18	4.4%

## Discussion

With the goal of enhancing awareness and education on iodine deficiency disorders (IDDs), our objective was to evaluate the comprehension of iodine's significance and the role of iodized salt in sustaining adequate iodine levels among adults aged 18 to 65 in the UAE. Notably, iodine deficiency problems in the UAE have diminished since 1994, with IDDs prevalence ranging from mild to normal across the country [5]. A study conducted in 2021 assessing urinary mineral excretion among primary school children in the emirate of Dubai revealed that only 10% of the Emirati population exhibited deficient iodine levels [6]. These findings are based on the median urinary iodine concentrations following the guidelines of the WHO [7,8]. This decline in IDD rates is primarily credited to salt iodization, as evidenced by the mandated legalization of salt fortification in 2007 in alignment with the standards set by the Gulf Corporation Council Standardization Organization (GCC GSO) [9]. Our study results revealed a few significant points about the perceptions of UAE residents regarding iodized salt, while there is a moderate level of awareness and understanding about iodine itself, there remains a significant lack of understanding about salt iodization, its benefits, and its importance in daily consumption. Yet, a substantial portion of participants continue to choose iodized salt for purchase and demonstrate appropriate storage practices for it.

In our study, participants with higher levels of education displayed a notable advantage in their understanding of iodine compared to those with lower educational backgrounds, as indicated by a calculated knowledge score. Interestingly, age did not influence the level of knowledge about iodine. These results are consistent with a study conducted in India, which similarly found a positive association between higher education levels and a deeper comprehension of iodine [10]. Conversely, another study conducted in Saudi Arabia found that knowledge levels were not influenced by increased educational levels [11]. These observations emphasize the significance of education in augmenting awareness about iodine and its importance. On the other hand, our study found that only half of the respondents were familiar with iodized salt. Interestingly, those who were aware of iodized salt tended to be older, between the ages of 35 and 65, and had higher levels of education. We speculate that this trend may possibly stem from older individuals, often managing household affairs, and being more proactive in educating themselves about healthier grocery options. A worth noting observation is that a comparative study in Ethiopia [12] indicated significantly higher awareness levels, with 80% of participants having good knowledge of iodized salt.

Iodine is essential for maintaining thyroid health as it enhances the production of thyroid hormones. Furthermore, iodine deficiency stands as one of the leading causes of goiter globally [13]. The implementation of salt iodization notably contributed to a decline in the prevalence of thyroid gland enlargement in the UAE, decreasing from 40.4% in 1994 to 8.2% in 2009 [14]. Similar trends were observed in the 20th-century United States, where thyroid size significantly diminished [15]. Nonetheless, our study uncovered only a modest level of understanding among participants regarding the importance of iodine for thyroid function. Furthermore, there was a moderate awareness regarding iodine sources, with only about half of the participants recognizing seafood as a good source of iodine. A comparable study conducted in Australia targeting iodine awareness among pregnant women yielded similar findings, with only 50-58% of women identifying seafood as a source of iodine [16].

Our study delved into participants' perspectives on iodized salt, exploring its advantages, cost, and recommended consumption compared to non-iodized salt. Participants exhibited a moderate level of awareness regarding the health benefits of iodized salt and held diverse views regarding its dietary significance. However, a clear trend emerged: UAE residents generally favor iodized salt over non-iodized options. Despite this preference, a considerable number expressed uncertainty about the appropriate quantity to incorporate into their daily diets. According to the WHO, adults should strive for a daily iodine intake of 150 µg, achievable through consuming just half to a quarter teaspoon of iodized salt daily, provided it contains the recommended international percentage of 15-40 mg/kg salt [17]. The salt fortification standards in the UAE maintain a concentration of 27.5 mg/kg of salt, aligning with the guidelines set by the GCC GSO and in accordance with WHO recommendations [9]. Nevertheless, our research uncovered a worrying trend of inconsistent iodized salt usage. Among those who purchase iodized salt, only 26% reported using it on a daily basis, while others exhibited varying usage frequencies, ranging from five to six days a week to as infrequent as once or twice a month, such inconsistent usage may not adequately meet the dietary requirements for iodine.

Iodized salt preserves higher levels of iodine when stored in optimal conditions, specifically in a cooler environment and with lower humidity levels, compared to environments with higher humidity levels, according to a study conducted in China, iodized salt stored at 37°C and 78% humidity experienced a decrease in iodine levels of approximately 18-28% [18]. In our study, participants exhibited positive practices in handling iodized salt. A significant majority place their salt in locations shielded from sunlight, safeguarding it from heat and light and ensuring its stability, by using closed containers when storing. Despite these responsible habits, nearly half of our participants were uncertain about their salt storage duration period, and only 29% stored their salt for a duration of less than two months which is a figure lower than a similar study conducted in Bangladesh where 67.5% of respondents stored their iodized salt for less than two months [19]. Another comparable study in India revealed that 85.5% of families stored it for up to 30 days only, while the remaining 14.5% stored it for more than 30 days [20]. Storing iodized salt for shorter durations is preferable, as evidenced by the literature, studies have shown that salt tends to lose its iodine content when stored for longer durations, regardless of the storage method [21]. Another study conducted in Ethiopia shows that iodized salt stored for less than two months contained 1.6 times higher iodine levels compared to salt stored for longer periods [22].

To our knowledge, this study is the first study to assess the knowledge, attitudes, and practices of the UAE population regarding iodized salt, it serves as a crucial initiative aimed at enhancing awareness regarding IDD and emphasizing the significance of iodized salt consumption. Notably, there has been a lack of recent studies assessing IDD levels in the country, with the last comprehensive study dating back to 1994. This absence presents a significant limitation, as understanding the current landscape is imperative for addressing IDD effectively. Furthermore, assessing the effectiveness of iodization practices in recent years is crucial for making informed policy decisions and implementing targeted public health interventions, it is hoped that this study will inspire further research in this area. Despite its contributions, this study faced several limitations, one significant challenge was reaching rural and remote areas in the country, specifically Ras Al-Khaimah and Umm Al Quwain, impacting the comprehensiveness of our results. These regions are densely populated and vital for a comprehensive study, but due to accessibility issues, our sample size from these areas remained limited. Additionally, our survey was self-administered, introducing the possibility of participants misinterpreting certain questions, which could have influenced the accuracy of the responses.

## Conclusions

As the UAE advances toward its goal of becoming a global hub, the health of its population remains fundamental to sustainable development. The investigation into iodized salt knowledge among the UAE population reveals a significant knowledge gap that could impact public health outcomes. Despite commendable efforts to raise awareness about iodized salt through governmental initiatives, our study highlights a persistent lack of understanding among participants. Only half of the participants are aware of iodized salt, its significance, benefits for the thyroid gland, and recommended daily intake. Furthermore, despite nearly half of the participants choosing to purchase iodized salt, our study reveals a concerning pattern of inconsistent iodized salt usage in the daily diet.

To address knowledge gaps identified in the study, there is a need for targeted awareness campaigns which could be conducted by integrating educational initiatives within healthcare settings and educational institutions disseminating accurate information about the importance of iodine, iodized salt, and iodine-rich foods. Continuous assessment and further research in this field are also essential to measure awareness and ensure sustained iodine sufficiency in the population.

## Appendices

**Table 6:** Knowledge, attitudes, and practices on the iodized salt questionnaire

Demographics	Answers
1. Gender	a. Male
	b. Female
2. Age (Years)	a. 18-24
	b. 25-34
	c. 35-44
	d. 45-54
	e. 55-65
3. Occupational status	a. Unemployed
	b. Part-time employment
	c. Full-time employment
	d. Housewife
	e. Retired
4. Education level	a. Didn’t complete elementary
	b. Elementary (Grades 1-6)
	c. Intermediate (Grades 7-9)
	d. Secondary (Grades 10-12)
	e. University
5. Emirates of residency	a. Abu Dhabi
	b. Dubai
	c. Sharjah
	d. Ajman
	e. Umm Al Quwain
	f. Ras Al Khaimah
	g. Fujairah
6. Marital status	a. Married
	b. Single
7. Cooking frequency	a. Always
	b. Usually (5-6 days/week)
	c. Often (3-4 days/week)
	d. Sometimes (1-2 days/week)
	e. Rarely (1-2 days/month)
	f. Never
	8. No. of residents in the household	a. Living Alone
b. 2 People	
c. 3 People	
d. 4 People	
e. 5 People	
f. More than 5 people (Specify____)	
Questions on knowledge about iodine	Answers
9. Have you ever heard of iodine?	a. Yes
	b. No
10. Which foods do you think contain iodine?	a. Fruits and vegetables
	b. Seafood
	c. Meat and poultry
	d. Dairy products
	e. I don’t know
	f. Others (Specify____)
	11. Do you know which part of the body needs iodine for proper function?	a. Yes
b. No	
12. If (Yes) which body part needs iodine to function?	a. Brain
	b. Muscles
	c. Thyroid gland
	d. Digestive system
	e. Heart
	f. Lungs
	g. Other (Specify____)
13. What do you think are the consequences of having low levels of iodine in your body?	a. Slow growth
	b. Mental damage
	c. Physical damage
	d. I don’t know
Questions on knowledge about Iodized salt	Answers
14. Do you know what iodized salt is?	a. Yes
	b. No
	c. I’m not sure
15. Do you think iodized salt is better than regular salt?	d. Much worse
	e. Somewhat worse
	f. About the same
	g. Somewhat better
	h. Much better
16. Do you think iodized salt is beneficial?	a. Yes
	b. No
	c. I don’t know
17. If (yes) what are the benefits of iodized salt?	a. Prevents thyroid Problems
	b. Improves overall health
	c. Improves pregnancy outcomes
	d. Mental and physical growth
	e. I don’t know
	f. I don’t think it’s beneficial
	g. Other (Specify____)
18. How much iodized salt do you think should be consumed in comparison to regular salt?	a. Iodized salt should be consumed more
	b. Iodized salt should be consumed less
	c. Iodized salt should be consumed in similar amounts of regular salt
	d. I don’t know
19. How do you think you can prevent low levels of iodine in your body?	a. Iodized salt
	b. Iodine supplements
	c. Seafood
	d. I don’t know
	e. Other (Specify____)
20. Do you think iodized salt is worth its price?	a. Yes
	b. No
	c. I don’t know
21. How important do you think iodized salt is in your diet?	a. Not at all important
	b. Low importance
	c. Slightly important
	d. Neutral
	e. Moderately important
	f. Very important
	g. Extremely important
Questions on attitudes about Iodized salt	Answers
22. What kind of salt do you buy?	a. Iodized salt
	b. Non-iodized salt
	c. I don’t know
23. If yes, why would you buy iodized salt?	a. I know it’s beneficial
	b. Other kinds of salt aren’t available
	c. I was advised to buy it
	d. Other reasons (Specify___)
24. If not, why would you not buy iodized salt?	a. I don’t know why it is important
	b. It is more expensive
	c. Other reasons (Specify___)
25. Do you have any concerns about adding iodized salt to your diet?	a. Yes
	b. No
	c. I’m not sure
Questions on practices of iodized salt	Answers
26. How often do you use iodized salt?	a. Always
	b. Usually (5-6 days/week)
	c. Often (3-4 days/week)
	d. Sometimes (1-2 days/week)
	e. Rarely (1-2 days/month)
	f. Never/I don’t know if I use iodized salt
27. For how long do you store iodized salt?	a. Less than 2 months
	b. More than 2 months
	c. I’m not sure
28. Where do you store iodized salt?	a. Cabinets
	b. Drawer
	c. Fridge
	d. Outside the kitchen
	e. I’m not sure
	f. Other places (Specify_____)
29. Is the storage place exposed to sunlight?	a. Yes
	b. No
	c. I’m not sure
30. Do you store it in a closed container?	a. Yes
	b. No
	c. I’m not sure
